# All-sense-all networks are suboptimal for sensorimotor synchronization

**DOI:** 10.1371/journal.pone.0202056

**Published:** 2018-08-29

**Authors:** Arnout van de Rijt

**Affiliations:** Department of Sociology, Utrecht University, Utrecht, the Netherlands; Northwestern University, UNITED STATES

## Abstract

In human groups that seek to synchronize to a common steady beat, every member can typically perceive every other member. We question whether this naturally occurring all-sense-all condition is optimal for temporal coordination. We consider alternative configurations represented by directed graphs, in which individuals can only hear or see a subset of others. We identify a trade-off in the topology of such networks: While denser graphs provide stronger coupling, improving synchrony, density increases sensitivity to early taps, which produces rushing. Results from an experimental study with music conservatory students show that networks that combine short path length with low density match all-sense-all networks in synchrony while yielding a steadier beat. These findings suggest that professional teams in arts, sports, industry, and the military may improve temporal coordination by employing technology that strategically configures who can track whom.

## Introduction

While rhythmic flashing and chorusing have been observed in a range of species [1–7], the ability to perceive a beat and synchronize body movement to it is unusually developed in humans [8–10]. Joint coordination on a common steady beat is a task human groups frequently face. The task appears in areas as diverse as musical performance, warfare, parades, air shows, rowing, synchronized sports, construction, production lines, acrobatics, traffic, and dance [10–17]. Successful temporal coordination involves minimizing (i) interpersonal timing differences (asynchrony) and (ii) fluctuations in tempo (arrhythmicity). In most instances of the phenomenon, conditions are such that each group member can hear or see every other member. Although nearby signals may be more salient, typically all members are influenced by all others in their attempts to coordinate on a rhythm. Here we probe whether this natural all-sense-all condition is optimal for temporal coordination. We consider the possibility of disabling sensory connections from some group members to others, which can be accomplished using physical barriers, headphones, visors, and so forth. Such incomplete patterns of sensory connectedness can be modeled as directed graphs [18–19], the topological effects of which can be studied.

We identify a trade-off in the effect of a network’s density–the number of ties as a fraction of all possible ties–on dimensions of temporal coordination. On the one hand, theoretical studies of synchronization in complex networks generally find that density, alongside other structural properties, improves synchrony in systems of coupled dynamical oscillators [20–23]. This suggests that all-can-sense-all networks may be optimal for minimizing interpersonal timing differences. On the other hand, empirical work on sensorimotor synchronization has established an important bias in human efforts at entrainment to a beat: People tend to adjust more to beats that come unexpectedly early than to those that come late [24–33]. This bias produces rushing when people mutually adjust their pace to one another’s previous tap. While rushing has not been found in animal systems [5, 7], it is a known phenomenon in bands and orchestras [12] and mild rushing has been reported in some experimental studies of mutual entrainment between two individuals [25, 27–28, 31–33]. We argue that network density will exacerbate rushing, namely by increasing the chance individuals perceive an accidental early tap by someone else. The resulting rushed responses are in turn broadcast more widely, producing yet more rushing. These expectations are consistent with a recent study that observed greater degrees of rushing with increasing group size [34], because large groups similarly expose their members to more early signals from other members than small groups.

Our argument raises the possibility that, counterintuitively, a team’s temporal coordination could be improved by reducing the number of others that members can simultaneously coordinate with. A topological feature critical to network synchronizability in many models is characteristic path length: the average length of the shortest path between any two nodes [20–22]. We hypothesize that decreasing density while keeping path length low will maintain small interpersonal timing differences while greatly reducing rushing.

## Materials & methods

To test this hypothesis, we conducted an experiment in which we systematically varied network topology by switching dyadic sensory connections on and off (see SI Appendix). The experiment was approved by the Stony Brook University Institutional Review Board (CORIHS# 2016-3451-F), after review of documents that included a signed letter of attestation stating that the experiment adheres to the ethical and legal requirements of the Central Committee on Research Involving Human Subjects (CCMO) of the Netherlands. We recruited students at the Amsterdam Conservatory into our experiment (18–25 years old, 71% male), increasing the external validity of our setting with respect to professional teams. All subjects provided written informed consent. In each session, six subjects were each asked to sit behind a table with a piano keyboard on top. Seat assignment was random. Subjects wore headsets that were hooked up to a computer system that controlled who could hear whom. They were asked to start off tapping the C4 key at a pace of about 60 beats per minute and then attempt to synchronize with the other players they could hear through their headsets to the best of their ability. The tables were arranged in a circular formation, with the subjects facing outward, such that they could not see one another’s tapping. No metronome or click track was used as aid. After 90 seconds they were asked to stop. This task was repeated with 42 different network assignments.

**Fig 1:**
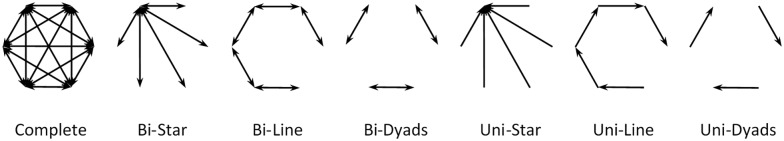
Topology of seven auditory networks tested in the experiment. Each arrow represents a source node being able to hear a destination node. Networks are sorted from left to right by decreasing density (see *SI Appendix*).

We assigned subjects to positions in seven network structures [35] with large variation in density and path length (Fig 1). Musicians could always hear themselves and these reflexive ties have been omitted from the Fig 1 networks for ease of interpretation. The default network is the Complete graph in which all can hear all. The Bi-Line and Bi-Star have the same density but vary in path length. The same holds for the Uni-Line and Uni-Star. The Uni-Dyads and Bi-Dyads structures allow isolation of synchronization behavior at the dyadic level. In each session, we studied six trials of each network structure, in each of which subjects were assigned to a different node, yielding a total of 90,143 taps.

**Fig 2:**
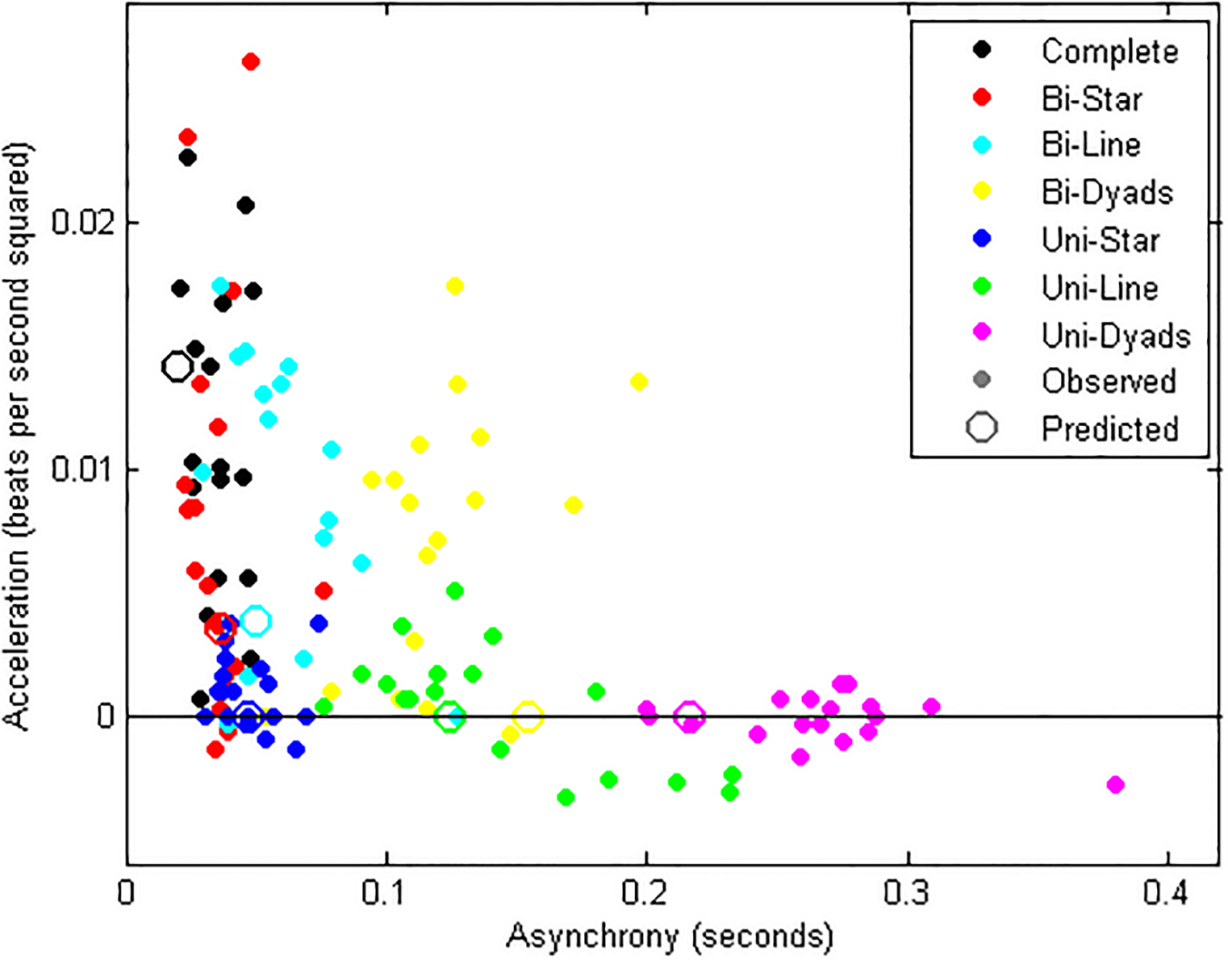
Beat-keeping performance of networked groups of conservatory students along two dimensions of temporal coordination. At the origin, asynchrony and acceleration are null, representing optimal coordination. Network structures are identified by distinct colors. For each network structure, observations from all 18 trials are shown (dots) as well as a prediction from the model given by Eqs (1) and (2) with parameters *α* = 0.5, *β* = 0.2, and *σ* = 0.035 (open circle).

We generalized the elementary measure of asynchrony most commonly used in the literature for pairs of mutually synchronizing humans–the average time difference between two taps from different subjects [25]–to larger groups, taking each tap’s temporal distance to the nearest tap from each other subject, averaged across taps and subjects. We calculated acceleration, the other dimension of temporal coordination, as the per second increase in pace over the course of the trial averaged across subjects. Fig 2 shows asynchrony by acceleration in each of the 18 trials for each of the seven network topologies.

## Results

Fig 2 reveals that network topology had a remarkably consistent effect on temporal coordination. The seven networks occupy distinct, well-defined regions in the two-dimensional space into which the rhythmic performance of different groups of subjects repeatedly fell. The Complete network combined minimal asynchrony with the greatest degree of acceleration. Rushing in the Complete network was so bad that in each trial of the experiment students expressed surprise and frustration with the group’s poor beat-keeping ability. Consistent with our hypothesis, we were able to identify networks in which rushing was reduced without sacrificing synchrony: The same students rushed significantly less in the sparser Bi-Star network (ranksum test, *p* < .05), while maintaining the same level of asynchrony (*p* > .05). In the even sparser Uni-Star, rushing was further reduced to a minimum (*p* < .001), while asynchrony grew only slightly, about a hundredth of a second, above the level in the Complete network (*p* < .05). This small increase in asynchrony need not be interpreted as a sacrifice for the much greater tempo steadiness that the Uni-Star network yields compared to the Complete network. Namely, it has been found that at slower speeds people find it slightly more difficult to synchronize [27]. Indeed, Fig 2 shows that asynchrony in the Uni-Star was comparable to trials with equally low levels of acceleration in the Complete network.

The dramatic increase in path length in the Bi-Line, Bi-Dyads, Uni-Line, and Uni-Dyads network had the anticipated effect of deteriorating synchrony vis-à-vis the Complete network (*p* < .001 in each). Specifically, the greater asynchrony observed in the Bi-Line and Uni-Line networks compared to the Bi-Star and Uni-Star networks is consistent with the assumption of path length negatively impacting synchrony, net of density. Uni-directional networks consistently exhibited better beat-keeping but worse synchrony than their bi-directional counterparts (*p* < .001 for Uni-Line vs. Bi-Line, Uni-Star vs. Bi-Star, and Uni-Dyads vs. Bi-Dyads).

The observed combinations of asynchrony and tempo change that characterize different network topologies are reasonably predicted from a commonly used model of linear phase and period updating [36–38], generalized from dyads to networks:

$${\Delta t}_{i,s+1}=(T_{i,s}+(\alpha +\beta )({\text{min}}_{j\in N_{i}}t_{j,s}-t_{i,s}))\varepsilon_{i,s+1}$$ (1)

$$T_{i,s+1}=T_{i,s}+\beta ({\text{min}}_{j\in N_{i}}t_{j,s}-t_{i,s})$$ (2)

In this model, individuals *i* infer phase and period changes after each tap *s*. They update their phase, realizing the inter-tap interval described by Eq (1), and update the period of their internal clock *T*_*i*,*s*_ following Eq (2). Both phase and period are updated in the direction of early taps, through addition of a portion *α*,*β* ∈ [0,1] of the time interval between *i*’s own tap *t*_*i*,*s*_ and the first tap among network neighbors *N*_*i*_ ⊆ {1, …, *i*-1, *i*+1, …, *N*}. Individuals make errors in timing following *ε*, which is log-normally distributed with mean 0 and standard deviation *σ*.

We obtained predictions for each network by simulating experimental trials using the general model given by Eqs (1) and (2). We explored each combination of parameter values *α*∈{0,0.1,…,1], *β*∈{0,0.1,…,1], and *σ*∈[0,0.01,…,0.05]. For a given combination we calculated each network’s average trial asynchrony and acceleration across 1,000 simulation runs. In order to assess model fit we needed to weigh errors in predicting asynchrony against errors in predicting acceleration. While any weighting is arguably arbitrary, we decided to weigh standardized scores equally, thus weighing inversely by the estimated standard deviation of the two variables. We thus defined predictive error as the sum of squared differences between predicted and observed normalized asynchrony scores and between predicted and observed normalized acceleration scores. Results are robust against modest changes in these weights. The model minimizing this sum of squared errors (at 144.7) across all 18 trials of all 7 networks has parameter settings *α* = 0.5, *β* = 0.2, and *σ* = 0.035. The trial averages predicted by this model (Fig 2 open circles) roughly identify the seven topological regions.

## Discussion

These findings go against the base assumption underlying the longstanding literature on coupled oscillators that greater coupling unequivocally improves temporal coordination. For individuals who tap in anticipation of others’ taps, increases in synchrony are counteracted by a loss of rhythmicity. The results confirm the surprising prediction derived here that the state of nature in human groups whereby all can perceive all is suboptimal for coordination on a steady pulse. Counterintuively, preventing people from being able to hear other members of the group can improve their ability to jointly synchronize to a common beat. The effects of graph topology on temporal coordination are remarkably well-defined, rendering these sensory networks ‘tunable’. We anticipate applications in arts, industry, sports, and the military wherever central beat-keeping aids such as a conductor, click track, captain, or sergeant are unavailable or impractical. In these settings, professional teams can improve temporal coordination by strategically employing technology that restricts who can track whom.

## Supporting information

S1 Appendix Experiment Details and Supplementary Analysis. (DOCX)
