# Supplementary material for: All-sense-all networks are suboptimal for sensorimotor synchronization
Source: PLoS One. 2018 Aug 29;13(8):e0202056. doi: 10.1371/journal.pone.0202056 (PMC6114297; doi:10.1371/journal.pone.0202056)
Supplement: S1 Appendix — (DOCX) [file pone.0202056.s001.docx]

Supporting Information for

**All-Sense-All Networks are Suboptimal for Sensorimotor Synchronization**

Experimental Design

The experiment was approved by the Stony Brook University Institutional Review Board (CORIHS# 2016-3451-F) and adheres to the ethical and legal requirements of the Central Committee on Research Involving Human Subjects (CCMO) of the Netherlands, as formally attested by Prof. Dr. Ineke Maas of Utrecht University, the Netherlands. The study was conducted in a rehearsal room at the Amsterdam Conservatory on Friday May 27, 2016 and Friday June 10, 2016. On each day 2 sessions were completed, with 6 subjects per session. All 24 subjects participated only once. Subjects were recruited by the program director using internal email. There were no inclusion criteria other than enrollment in the program, which means all subjects had passed the Amsterdam Conservatory entrance examination, having demonstrated the ability to distinguish between and name all intervals, scales and triads, sight-read a melody, tap a rhythm, write down a short melodic or rhythmic piece, read treble and bass clefs, and perform an original composition of sufficient quality. Subjects’ ages ranged from 18 to 25 years old, and 71% were male. Subjects were paid 50 Euros in cash for participation. Sessions lasted between one-and-a-half and 2 hours. The experiment was conducted in the English language, which all subjects commanded.

In the experimental setup, six tables were arranged in a circular formation facing outward, with a regular 60° angle between each pair of neighboring tables. A keyboard and a headset were placed on each table. Both were connected to a computer system that determined who could hear whom. The computer system was controlled by a professional studio technician. During each of the sessions the technician iterated through six permutations of each of the seven study networks shown in Figure 1 in the main text, for a total of 42 trials. Topological features of the networks are shown in Table S1. Adjacency matrices of the permuted networks used in each of the 42 trials are shown in Figure S1. The order in which the networks were superimposed on the participants was the same in sessions 1 and 3 and follows the order shown in Figure S1 from left to right, top to bottom. In sessions 2 and 4 the reverse order was used for counterbalancing.

At the beginning of each session, subjects upon arrival read and signed an informed consent form containing instructions. Subjects then drew a card from a shuffled deck containing six cards numbered 1 through 6 and sat down at a table displaying a matching musician number. Once all subjects were seated at the correct tables and were wearing their headsets the experimenter repeated the written instructions out loud. Each trial was initiated by the experimenter informing participants on the structure of the network. For example, prior to each trial with a “Bi-star” configuration, the experimenter told subjects:

“Musician 2 will be able to hear him/herself and everyone else. Musicians 1, 3, 4, 5 and 6 will only be able to hear themselves and one other musician”

The experimenter then asked subjects upon hearing the word “start” to start pressing the C4 key on their keyboard at a pace of about 60 beats per minute and then to the best of their ability attempt to synchronize with the other players they could hear through their headsets. No metronome or click track was used as aid. After 90 seconds, they were asked to stop after hearing the experimenter say the word “stop”.

The main text reports on three of the four sessions we conducted. We omitted the second session because in this session one of the subjects curiously tapped on off-beats, apparently maximizing the distance between his own tap and those of the other subjects he could hear. We had no choice but to deviate from script and instruct the subject to try to synchronize instead of tapping off-beats. The subject persisted with this curious behavior, resorting to the expected behavior only after several corrective statements by the experimenter. Analyses including this session are consistent with the results reported in the main text.

| Network ID | Network label | Density | Average path length  (in undirected graph) |
| --- | --- | --- | --- |
| A | Complete | 1 | 1 |
| B | Bi-Star | 1/3 | 1.8 |
| C | Bi-Line | 1/3 | 2.3 |
| D | Bi-Dyads | 1/5 | - |
| E | Uni-Star | 1/6 | 1.8 |
| F | Uni-Line | 1/6 | 2.3 |
| G | Uni-Dyads | 1/10 | - |

Table S1. Topological features of the study networks.

Analysis in main text

To evaluate effects of sensory network topology on asynchrony and acceleration, all tests reported in the main text compare trial-level measures between 18 trials of one network and 18 trials of another network. Rank-sum tests (*N* = 36) were used to obtain significance levels for these comparisons.

Subject differences

To test whether subjects differed in their degrees of asynchrony and acceleration, we calculated subject-level averages and confidence intervals for both quantities. Figure S2 shows that both within and across trials subjects showed significantly different levels of asynchrony. In session 1, the subjects seated behind keyboards 1 and 2 were not as synchronous as subjects behind the other four keyboards. In session 2, subjects 5 and 6 performed worse than the others. Subjects in session 3 all performed better on synchrony than subjects in the other two sessions. Despite their statistical significance, these differences are substantively small, ranging between 58 hundredths and 79 milliseconds. As Figure 2 in the main text shows, network topology had a much greater effect on synchrony than subjects’ identities or the session they were in.

Figure S3 shows that subject differences in acceleration were exclusively driven by the sessions they were in. This shows the lack of control individual subjects had over group tempo. Sessions 1 and 4 exhibited far greater acceleration than session 3. These differences are explained by great variability in the degree of acceleration in the Complete and Bi-Star networks, as Figure 2 already showed. In all other networks, the spread of acceleration across sessions and permutations was much smaller.

A: Complete

|  |  |  |  |  |  |
| --- | --- | --- | --- | --- | --- |
|  |  |  |  |  |  |
|  |  |  |  |  |  |
|  |  |  |  |  |  |
|  |  |  |  |  |  |
|  |  |  |  |  |  |

|  |  |  |  |  |  |
| --- | --- | --- | --- | --- | --- |
|  |  |  |  |  |  |
|  |  |  |  |  |  |
|  |  |  |  |  |  |
|  |  |  |  |  |  |
|  |  |  |  |  |  |

|  |  |  |  |  |  |
| --- | --- | --- | --- | --- | --- |
|  |  |  |  |  |  |
|  |  |  |  |  |  |
|  |  |  |  |  |  |
|  |  |  |  |  |  |
|  |  |  |  |  |  |

|  |  |  |  |  |  |
| --- | --- | --- | --- | --- | --- |
|  |  |  |  |  |  |
|  |  |  |  |  |  |
|  |  |  |  |  |  |
|  |  |  |  |  |  |
|  |  |  |  |  |  |

|  |  |  |  |  |  |
| --- | --- | --- | --- | --- | --- |
|  |  |  |  |  |  |
|  |  |  |  |  |  |
|  |  |  |  |  |  |
|  |  |  |  |  |  |
|  |  |  |  |  |  |

|  |  |  |  |  |  |
| --- | --- | --- | --- | --- | --- |
|  |  |  |  |  |  |
|  |  |  |  |  |  |
|  |  |  |  |  |  |
|  |  |  |  |  |  |
|  |  |  |  |  |  |

B: Bi-Star

|  |  |  |  |  |  |
| --- | --- | --- | --- | --- | --- |
|  |  |  |  |  |  |
|  |  |  |  |  |  |
|  |  |  |  |  |  |
|  |  |  |  |  |  |
|  |  |  |  |  |  |

|  |  |  |  |  |  |
| --- | --- | --- | --- | --- | --- |
|  |  |  |  |  |  |
|  |  |  |  |  |  |
|  |  |  |  |  |  |
|  |  |  |  |  |  |
|  |  |  |  |  |  |

|  |  |  |  |  |  |
| --- | --- | --- | --- | --- | --- |
|  |  |  |  |  |  |
|  |  |  |  |  |  |
|  |  |  |  |  |  |
|  |  |  |  |  |  |
|  |  |  |  |  |  |

|  |  |  |  |  |  |
| --- | --- | --- | --- | --- | --- |
|  |  |  |  |  |  |
|  |  |  |  |  |  |
|  |  |  |  |  |  |
|  |  |  |  |  |  |
|  |  |  |  |  |  |

|  |  |  |  |  |  |
| --- | --- | --- | --- | --- | --- |
|  |  |  |  |  |  |
|  |  |  |  |  |  |
|  |  |  |  |  |  |
|  |  |  |  |  |  |
|  |  |  |  |  |  |

|  |  |  |  |  |  |
| --- | --- | --- | --- | --- | --- |
|  |  |  |  |  |  |
|  |  |  |  |  |  |
|  |  |  |  |  |  |
|  |  |  |  |  |  |
|  |  |  |  |  |  |

C: Bi-Line

|  |  |  |  |  |  |
| --- | --- | --- | --- | --- | --- |
|  |  |  |  |  |  |
|  |  |  |  |  |  |
|  |  |  |  |  |  |
|  |  |  |  |  |  |
|  |  |  |  |  |  |

|  |  |  |  |  |  |
| --- | --- | --- | --- | --- | --- |
|  |  |  |  |  |  |
|  |  |  |  |  |  |
|  |  |  |  |  |  |
|  |  |  |  |  |  |
|  |  |  |  |  |  |

|  |  |  |  |  |  |
| --- | --- | --- | --- | --- | --- |
|  |  |  |  |  |  |
|  |  |  |  |  |  |
|  |  |  |  |  |  |
|  |  |  |  |  |  |
|  |  |  |  |  |  |

|  |  |  |  |  |  |
| --- | --- | --- | --- | --- | --- |
|  |  |  |  |  |  |
|  |  |  |  |  |  |
|  |  |  |  |  |  |
|  |  |  |  |  |  |
|  |  |  |  |  |  |

|  |  |  |  |  |  |
| --- | --- | --- | --- | --- | --- |
|  |  |  |  |  |  |
|  |  |  |  |  |  |
|  |  |  |  |  |  |
|  |  |  |  |  |  |
|  |  |  |  |  |  |

|  |  |  |  |  |  |
| --- | --- | --- | --- | --- | --- |
|  |  |  |  |  |  |
|  |  |  |  |  |  |
|  |  |  |  |  |  |
|  |  |  |  |  |  |
|  |  |  |  |  |  |

D: Bi-Dyads

|  |  |  |  |  |  |
| --- | --- | --- | --- | --- | --- |
|  |  |  |  |  |  |
|  |  |  |  |  |  |
|  |  |  |  |  |  |
|  |  |  |  |  |  |
|  |  |  |  |  |  |

|  |  |  |  |  |  |
| --- | --- | --- | --- | --- | --- |
|  |  |  |  |  |  |
|  |  |  |  |  |  |
|  |  |  |  |  |  |
|  |  |  |  |  |  |
|  |  |  |  |  |  |

|  |  |  |  |  |  |
| --- | --- | --- | --- | --- | --- |
|  |  |  |  |  |  |
|  |  |  |  |  |  |
|  |  |  |  |  |  |
|  |  |  |  |  |  |
|  |  |  |  |  |  |

|  |  |  |  |  |  |
| --- | --- | --- | --- | --- | --- |
|  |  |  |  |  |  |
|  |  |  |  |  |  |
|  |  |  |  |  |  |
|  |  |  |  |  |  |
|  |  |  |  |  |  |

|  |  |  |  |  |  |
| --- | --- | --- | --- | --- | --- |
|  |  |  |  |  |  |
|  |  |  |  |  |  |
|  |  |  |  |  |  |
|  |  |  |  |  |  |
|  |  |  |  |  |  |

|  |  |  |  |  |  |
| --- | --- | --- | --- | --- | --- |
|  |  |  |  |  |  |
|  |  |  |  |  |  |
|  |  |  |  |  |  |
|  |  |  |  |  |  |
|  |  |  |  |  |  |

E: Uni-Star

|  |  |  |  |  |  |
| --- | --- | --- | --- | --- | --- |
|  |  |  |  |  |  |
|  |  |  |  |  |  |
|  |  |  |  |  |  |
|  |  |  |  |  |  |
|  |  |  |  |  |  |

|  |  |  |  |  |  |
| --- | --- | --- | --- | --- | --- |
|  |  |  |  |  |  |
|  |  |  |  |  |  |
|  |  |  |  |  |  |
|  |  |  |  |  |  |
|  |  |  |  |  |  |

|  |  |  |  |  |  |
| --- | --- | --- | --- | --- | --- |
|  |  |  |  |  |  |
|  |  |  |  |  |  |
|  |  |  |  |  |  |
|  |  |  |  |  |  |
|  |  |  |  |  |  |

|  |  |  |  |  |  |
| --- | --- | --- | --- | --- | --- |
|  |  |  |  |  |  |
|  |  |  |  |  |  |
|  |  |  |  |  |  |
|  |  |  |  |  |  |
|  |  |  |  |  |  |

|  |  |  |  |  |  |
| --- | --- | --- | --- | --- | --- |
|  |  |  |  |  |  |
|  |  |  |  |  |  |
|  |  |  |  |  |  |
|  |  |  |  |  |  |
|  |  |  |  |  |  |

|  |  |  |  |  |  |
| --- | --- | --- | --- | --- | --- |
|  |  |  |  |  |  |
|  |  |  |  |  |  |
|  |  |  |  |  |  |
|  |  |  |  |  |  |
|  |  |  |  |  |  |

F: Uni-Line

|  |  |  |  |  |  |
| --- | --- | --- | --- | --- | --- |
|  |  |  |  |  |  |
|  |  |  |  |  |  |
|  |  |  |  |  |  |
|  |  |  |  |  |  |
|  |  |  |  |  |  |

|  |  |  |  |  |  |
| --- | --- | --- | --- | --- | --- |
|  |  |  |  |  |  |
|  |  |  |  |  |  |
|  |  |  |  |  |  |
|  |  |  |  |  |  |
|  |  |  |  |  |  |

|  |  |  |  |  |  |
| --- | --- | --- | --- | --- | --- |
|  |  |  |  |  |  |
|  |  |  |  |  |  |
|  |  |  |  |  |  |
|  |  |  |  |  |  |
|  |  |  |  |  |  |

|  |  |  |  |  |  |
| --- | --- | --- | --- | --- | --- |
|  |  |  |  |  |  |
|  |  |  |  |  |  |
|  |  |  |  |  |  |
|  |  |  |  |  |  |
|  |  |  |  |  |  |

|  |  |  |  |  |  |
| --- | --- | --- | --- | --- | --- |
|  |  |  |  |  |  |
|  |  |  |  |  |  |
|  |  |  |  |  |  |
|  |  |  |  |  |  |
|  |  |  |  |  |  |

|  |  |  |  |  |  |
| --- | --- | --- | --- | --- | --- |
|  |  |  |  |  |  |
|  |  |  |  |  |  |
|  |  |  |  |  |  |
|  |  |  |  |  |  |
|  |  |  |  |  |  |

G: Uni-Dyads

|  |  |  |  |  |  |
| --- | --- | --- | --- | --- | --- |
|  |  |  |  |  |  |
|  |  |  |  |  |  |
|  |  |  |  |  |  |
|  |  |  |  |  |  |
|  |  |  |  |  |  |

|  |  |  |  |  |  |
| --- | --- | --- | --- | --- | --- |
|  |  |  |  |  |  |
|  |  |  |  |  |  |
|  |  |  |  |  |  |
|  |  |  |  |  |  |
|  |  |  |  |  |  |

|  |  |  |  |  |  |
| --- | --- | --- | --- | --- | --- |
|  |  |  |  |  |  |
|  |  |  |  |  |  |
|  |  |  |  |  |  |
|  |  |  |  |  |  |
|  |  |  |  |  |  |

|  |  |  |  |  |  |
| --- | --- | --- | --- | --- | --- |
|  |  |  |  |  |  |
|  |  |  |  |  |  |
|  |  |  |  |  |  |
|  |  |  |  |  |  |
|  |  |  |  |  |  |

|  |  |  |  |  |  |
| --- | --- | --- | --- | --- | --- |
|  |  |  |  |  |  |
|  |  |  |  |  |  |
|  |  |  |  |  |  |
|  |  |  |  |  |  |
|  |  |  |  |  |  |

|  |  |  |  |  |  |
| --- | --- | --- | --- | --- | --- |
|  |  |  |  |  |  |
|  |  |  |  |  |  |
|  |  |  |  |  |  |
|  |  |  |  |  |  |
|  |  |  |  |  |  |

Figure S1. Adjacency matrices used in the 42 trials conducted in each session. Each matrix has 6 rows and 6 columns representing the 6 musicians. For each black cell in the matrix, row could hear column. For each white cell, row could not hear column.


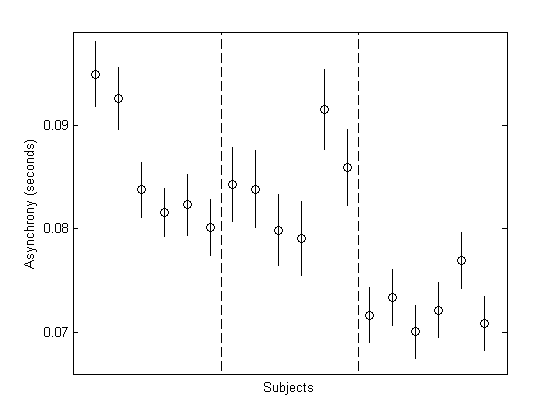


Figure S2. Average asynchrony of each subject. Solid lines represent 95% confidence intervals around subject means. Dashed lines separate subjects from different sessions.


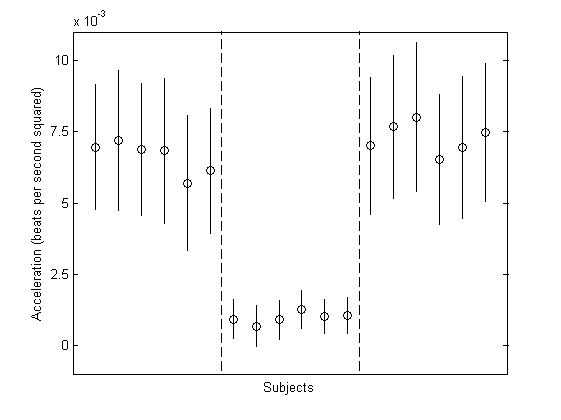


Figure S3. Average acceleration exhibited by each subject. Solid lines represent 95% confidence intervals around subject means. Dashed lines separate subjects from different sessions.
